# Toward a smart skin: Harnessing cuticle biosynthesis for crop adaptation to drought, salinity, temperature, and ultraviolet stress

**DOI:** 10.3389/fpls.2022.961829

**Published:** 2022-07-25

**Authors:** Lang Liu, Xiaoyu Wang, Cheng Chang

**Affiliations:** College of Life Sciences, Qingdao University, Qingdao, China

**Keywords:** cuticle, drought, salinity, extreme temperatures, ultraviolet radiation, crop improvement

## Abstract

Drought, salinity, extreme temperatures, and ultraviolet (UV) radiation are major environmental factors that adversely affect plant growth and crop production. As a protective shield covering the outer epidermal cell wall of plant aerial organs, the cuticle is mainly composed of cutin matrix impregnated and sealed with cuticular waxes, and greatly contributes to the plant adaption to environmental stresses. Past decades have seen considerable progress in uncovering the molecular mechanism of plant cutin and cuticular wax biosynthesis, as well as their important roles in plant stress adaptation, which provides a new direction to drive strategies for stress-resilient crop breeding. In this review, we highlighted the recent advances in cuticle biosynthesis in plant adaptation to drought, salinity, extreme temperatures, and UV radiation stress, and discussed the current status and future directions in harnessing cuticle biosynthesis for crop improvement.

## Introduction

Growing population and consumption greatly increase the global crop demand. It is expected that 70% more food is needed to feed 10 billion people by 2050 (Tilman et al., 2011). However, plant growth and global crop production are adversely affected by unfavorable environmental conditions such as water deficit (drought), salinity, extreme temperatures, and ultraviolet (UV) radiation. For instance, 10.1% loss in national cereal production was reported in drought years from 1964 to 2007 (Lesk et al., 2016). Notably, drought stress led to 20.6 and 39.3% yield reduction in wheat and maize, respectively between 1980 and 2015 (Daryanto et al., 2016; Fahad et al., 2017). As an environmental factor causing land degradation, salinity affects about 11% of the global irrigated areas and could lead to above 50% yield loss in salt-sensitive crop species including all important glycophytic crops (Zörb et al., 2019). During the past century, extreme temperatures have become more frequent and seriously affected agriculture production. It was estimated that global cereal production in extreme heat years was reduced by 9.1% on average during 1964–2007 (Lesk et al., 2016). Low-temperature stresses such as chilling (0–18°C) and freezing (<0°C) temperature significantly affect spatial distribution and production in cold-sensitive crop species like rice (Shimono et al., 2007; Wang P. et al., 2019; Zhang et al., 2019a). Similarly, increased UV radiation could induce morphological changes, alter genome stability and affect yields in sensitive crop species (Robson et al., 2019). In addition, stratospheric ozone depletion and global climate change have contributed to the increased incidence and prolonged duration of these environmental stresses during past decades. Therefore, developing crop varieties adapted to drought, salinity, extreme temperatures, and UV radiation stress is essential to secure and enhance food production.

As the true interface between plant aerial parts and surrounding environments, lipophilic cuticle synthesized by plant epidermal cells protects plant tissue from environmental stresses such as desiccation, extreme temperatures, increased UV radiation, pathogen infections, and mechanical damages, and greatly contributes to the plant adaptation to stressful terrestrial habitats (Domínguez et al., 2011; Kong et al., 2020a). Although cuticle composition varies among plant species, organs, developmental stages and environmental conditions, the cuticle is generally composed of lipids, polysaccharides and phenolic compounds (Yeats and Rose, 2013). Cuticular lipid components mainly consist of wax, cutin, and cutan polymers, which confer cuticle the hydrophobic property. As the framework of the plant cuticle, cutin polymers contain a large amount of cross-linked polyester of oxygenated long-chain (C_16_ or C_18_) fatty acids and their derivatives (Philippe et al., 2020). Cuticular wax mixtures are mainly composed of very-long-chain (VLC, >C_20_) fatty acids, alcohols, aldehydes, alkanes, esters, and ketones (Lee and Suh, 2015; Wang et al., 2020; Suh et al., 2022). Compared with cutin and wax, cutan polymers are less explored in structure due to their non-hydrolysable bounds (Bhanot et al., 2021; Reynoud et al., 2021). Phenolic compounds identified in cuticle fraction include hydroxycinnamic acids such as ð-coumaric, ferulic, caffeic, and ð-hydroxybenzoic acids, as well as flavonoids in some species (Fernández et al., 2016). As an adaptive innovation in land plants, lipophilic cuticle could protect plant tissues from environmental stresses, thereby gaining increasing attention in the study of plant stress tolerance (Li and Chang, 2021). Herein, we summarized the most recent progress in studies of cuticle biosynthesis in plant adaptation to drought, salinity, extreme temperatures, and UV radiation stress, and discussed the potentials, challenges and strategies in exploiting cuticle biosynthesis for crop improvement.

## Cuticle biosynthesis in model and crop plants

With the contribution of cuticle biosynthetic mutants and advanced cuticle phenotyping methods, cuticle biosynthetic pathways are extensively studied in the model plant *Arabidopsis thaliana* (Yeats and Rose, 2013; Suh et al., 2022). As summarized in previous reviews, cuticular wax and cutin are synthesized mainly by modification of plastid-derived C16 and C18 fatty acids in the endoplasmic reticulum (ER) of plant epidermal cells (Philippe et al., 2020; Wang et al., 2020). For the cutin monomer biosynthesis, plastid C16 and C18 fatty acids were conjugated to coenzyme A (CoA) by long-chain acyl-coenzyme A synthases (LACS) and then trafficked to the ER, where the C16 and C18 acyl-CoAs could undergo aliphatic chain elongation into C20–C26 acyl-CoAs catalyzed by the fatty acid elongase (FAE) enzyme complex consisting of β-ketoacyl-CoA synthases (KCS), β-ketoacyl-CoA reductase (KCR), 3-hydroxyacyl-CoA dehydratases (HCD), and enoyl-CoA reductases (ECR) (Yeats and Rose, 2013; Fich et al., 2016; Philippe et al., 2020). These C16–C26 acyl-CoAs are oxidized at their terminal and/or midchain carbons by cytochrome P450 enzymes (CYP77 and CYP86) and the protein HOTHEAD, hydroxylated by epoxide hydrolases (EH), and finally modified into monoacylglycerol cutin monomers by glycerol-3-phosphate acyltransferases (GPAT) (Pineau et al., 2017; Philippe et al., 2020). At the same time, the phenolic compound ferulic acid converted from coumaric acid by cytochrome P450 enzyme (CYP98) is conjugated to monoacylglycerol cutin monomers under the action of the transferase enzyme DEFICIENT IN CUTIN FERULATE (DCF) (Fich et al., 2016; Philippe et al., 2020). These cutin monomers are then exported out of the cell via the plasma membrane (PM)-localized ABC type of transporters and deposited into the cuticle, where cutin synthase (CUS) proteins mediate the cutin polymerization (Fich et al., 2016; Hong et al., 2017).

For the cuticular wax biosynthesis, plastid-derived C16 and C18 acyl-CoAs were first elongated to VLC (up to C34) acyl-CoAs by the FAE complex and the ECERIFERUM2 (CER2) protein (Haslam et al., 2015). It was recently demonstrated that protein-protein interactions in the FAE complex, including extensive protein–protein interactions among *Arabidopsis* FAE complex proteins KCR1, PASTICCINO2 (PAS2), ECR, and PAS1, as well as specific interactions between KCS9 and PAS2 or ECR, are essential to the VLC acyl-CoAs elongation (Kim et al., 2021). These elongated VLC acyl-CoAs were then either converted into *n*-alkanes, *n*-aldehydes, secondary alcohols, and ketones via the alkane-forming pathway or modified into primary *n*-alcohols and esters through the alcohol-forming pathway. In the alkane-forming pathway, VLC acyl-CoAs were converted into VLC alkanes under the action of the ECERIFERUM1 (CER1)/ECERIFERUM3 (CER3)/CYTOCHROME B5 (CYTB5) complex, and then oxidized to secondary alcohols and ketones by the CYP95A family cytochrome P450 enzymes (MAH1) (Pascal et al., 2019; Tang et al., 2020). In the alcohol-forming pathway, VLC acyl-CoAs were converted into primary alcohols under actions of acyl desaturase ECERIFERUM17 (CER17) and fatty acyl-CoA reductase ECERIFERUM4 (CER4), and modified into wax esters by WAX SYNTHASE/ACYL-COA:DIACYLGLYCEROL ACYLTRANSFERASE 1 (WSD1) (Yang et al., 2017). These generated wax components such as VLC fatty acids, alcohols, aldehydes, alkanes, esters, and ketones were transported from the ER to the PM via the Golgi and *trans*-Golgi network (TGN)-trafficking pathways, and finally exported out of the plant cell to the cuticle via the PM-localized ATP binding cassette G (ABCG) subfamily half transporters and the lipid transfer proteins (LTPs) (Yeats and Rose, 2013; Lee and Suh, 2015; Suh et al., 2022). In addition to these straight-chain wax components, branched waxes such as iso-alkanes and iso-alcohols have been identified in *Arabidopsis* flowers and leaves, and are derived from the catabolism of branched-chain amino acid (BCAA) such as valine. The isobutyl-CoA synthetase ACYL-ACTIVATING ENZYME 9 (AAE9) was recently identified to connect the BCAA catabolism with branched wax biosynthesis in the plastid (Li et al., 2022).

Increasing evidence revealed that cuticle biosynthesis is tight regulated at transcriptional, post-transcriptional, and post-translational levels. For instance, *Arabidopsis* APETALA2-Ethylene responsive factor (AP2-EREBP)-type transcription factors (SHN1/2/3), myeloblastosis (MYB) family transcription factors (MYB16, MYB30, MYB41, MYB94, MYB96, MYB106), zinc-finger transcription factors (NFXL2), and the class IV homeodomain-leucine zipper family transcription factors (HDG1) widely get involved in the transcriptional regulation of cutin and cuticular wax biosynthesis (Yeats and Rose, 2013; Lee and Suh, 2015; Philippe et al., 2020; Suh et al., 2022). ECERIFERUM16 (CER16), RNA exosome subunit RRP45B and Ski complex components (SKI2, SKI3, SKI7, and SKI8) regulate the post-transcriptional gene silencing of *CER3*, a VLC fatty acid reductase gene (Lee and Suh, 2015; Yang X. et al., 2020). E3 ubiquitin ligases ECERIFERUM9 (CER9), HMG-CoA Reductase Degradation 1 (HRD1), SMALL AND GLOSSY LEAVES1 (SAGL1) and ABA-related RING-type E3 ligase (ARRE) contribute to the post-translational regulation of cuticular lipid biosynthesis (Zhao et al., 2014; Kim et al., 2019; Liu et al., 2021; Wu et al., 2021).

With the assistance of forward and reverse genetic approaches, past decades have seen great advances in functional characterization of genes essential for cuticle biosynthesis in many crop species such as *Zea mays*, *Triticum aestivum*, *Hordeum vulgare*, *Oryza sativa*, *Solanum lycopersicum*, *Glycine max*, *Malus domestica*, *Medicago truncatula*, *Camellia sinensis*, *Sorghum bicolor*, *Arachis hypogaea*, *Cyperus esculentus*, *Gossypium hirsutum*, *Citrus sinensis*, *Cucumis sativus*, and *Brassica napus* (Zhou et al., 2013; Wang et al., 2015; Zhou et al., 2015; Li et al., 2018, 2019; Lokesh et al., 2019; Cheng et al., 2020; Guo et al., 2020; Zhang et al., 2020a; Abdullah et al., 2021; Ayaz et al., 2021; Busta et al., 2021; Lu et al., 2021; Wu et al., 2022). Although cuticle composition varies among plant species, evolutionarily conserved functions were revealed in cuticle biosynthesis genes of model and crop plants. For instance, tomato cutin synthase (SlCUS1), together with its homolog in *Arabidopsis* and the moss *Physcomitrella patens*, exhibited cutin monomer polymerizing activity *in vitro* (Yeats et al., 2014). Expression of *GmLACS2-3*, soybean homolog of *Arabidopsis LACS2* gene, in the *Arabidopsis atlacs2* mutant could rescue its cutin-deficient phenotype (Ayaz et al., 2021). This functional conservation was also obvious for cuticular wax biosynthesis genes such as *CER1*, *CER2*, *CER3*, *ECR*, *KCR*, *KCS1*, *KCS2*, *KCS6* in model and crop plants. For instance, silencing the *SlCER1-1* gene, tomato homolog of *Arabidopsis AtCER1* gene, attenuated wax alkane biosynthesis in tomato (Wu et al., 2022). Consistent with this, ectopic expression of *MdKCS2* and *MdCER2*, apple homologs of *Arabidopsis AtKCS2* and *AtCER2* genes, in *Arabidopsis* could enhance the accumulation of cuticular wax in *Arabidopsis* leaves and stems (Zhong et al., 2020; Lian et al., 2021). Notably, the function of cuticular wax biosynthesis genes seems conserved among monocots and dicots. For instance, the mutation in *HvKCS1* and *HvKCS6*, barley homologs of *Arabidopsis AtKCS1* and *AtKCS6*, resulted in a substantial reduction in the total cuticular wax load in barley leaves (Weidenbach et al., 2014; Li et al., 2018). Similarly, reduced expression of *TaECR* and *TaKCS6*, wheat homologs of *Arabidopsis AtECR* and *AtKCS6*, was associated with significant reductions in total wax load in bread wheat (Wang X. et al., 2019; Kong et al., 2020b). In addition, the wheat nullisomic-tetrasomic substitution line lacking *TaCER1-1A* had significantly reduced amounts of C33 alkane (Li et al., 2019).

Recent studies revealed that regulatory genes in cuticle biosynthesis also showed functional conservation in model and crop plants. For instance, silencing *MdMYB30*, apple homologs of *Arabidopsis AtMYB30* gene, compromised wax crystal accumulation in apple (Zhang et al., 2019c). Similarly, tomato mutant with a mutation in *CUTIN DEFICIENT2* (*CD2*), tomato homolog of *Arabidopsis ANTHOCYANINLESS2* (*ANL2*), exhibited cutin deficiency in tomato fruit (Nadakuduti et al., 2012). Notably, this functional conservation of cuticle biosynthesis regulatory genes is also obvious between monocots and dicots. For instance, overexpression of *TaSHN1/WAX INDUCER1* (*TaWIN1*), wheat homologs of *Arabidopsis AtSHN1*, resulted in increased accumulation of wax alkanes in wheat leaves (Bi et al., 2018). Silencing wheat *TaWIN1* and barley *HvWIN1* could attenuate cuticular wax accumulation (Kong and Chang, 2018; McAllister et al., 2022). Similarly, overexpressing *OsWR1*, rice homologs of *Arabidopsis AtSHN1*, improved while silencing *OsWR1* attenuated wax biosynthesis in rice leaves (Wang et al., 2012).

## Cuticle biosynthesis in plant adaptation to drought stress

As the major transpiration barrier, the hydrophobic cuticle restricts the non-stomatal water loss and protects plant tissues from desiccation in drought conditions. Induction of cuticle biosynthesis, including activation of cuticle biosynthesis genes, by drought stress has been observed in a wide range of plant species such as *A. thaliana*, *O. sativa*, *T. aestivum*, *Z. mays*, *C. sativus*, *S. lycopersicum*, *M. domestica*, *S. bicolor*, *H. vulgare*, *G. max*, *G. hirsutum*, *C. sinensis*, and *G. hirsutum* (Islam et al., 2009; Weng et al., 2010; Zhou et al., 2013, 2015; Wang et al., 2015; Li et al., 2018, 2019; Lokesh et al., 2019; Guo et al., 2020; Zhang et al., 2020a; Abdullah et al., 2021; Ayaz et al., 2021; Lu et al., 2021; Wu et al., 2022). As summarized in Table 1, altered expression of cuticle biosynthesis genes such as *LACS1*, *LACS2*, *CER1*, *CER2*, *CER9*, *KCS1*, *KCS2*, *KCS6*, *FAR3.1*, *WSD1*, *GPAT2*, and *ABCG31* could lead to the changed drought tolerance in model and crop plants. Generally, over-expression of these cuticle biosynthesis genes results in the increased accumulation of cuticular wax or cutin, which contributes to the enhanced tolerance to drought stress. For instance, over-expression of *Arabidopsis* wax biosynthesis gene *AtCER1* and its homologs in wheat (*TaCER1-A*), cucumber (*CsCER1*) and tomato (*SlCER1-1*) could increase VLC alkanes accumulation and enhance drought tolerance in transgenic plants (Bourdenx et al., 2011; Wang et al., 2015; Li et al., 2018; Wu et al., 2022). Over-expression of *Arabidopsis* wax biosynthesis gene *AtWSD1* in *Arabidopsis* and *Camelina* resulted in enhanced drought tolerance in transgenic plants (Abdullah et al., 2021). Over-expression of *GmLACS2-3*, soybean (*Glycine max*) homolog of *Arabidopsis AtLACS2*, in *Arabidopsis* could enhance the amounts of cutin and suberin but not wax, and result in the increased drought tolerance (Ayaz et al., 2021). Consistent with the results of over-expression studies, knockout or knockdown of cuticle biosynthesis genes could lead to impaired cuticle development and decreased drought tolerance in model and crop plants. For instance, silencing of *SlCER1-1* in tomato reduced the amounts of n-alkanes and branched alkanes, and decreased plant drought resistance (Wu et al., 2022). Similarly, silencing of *GhFAR3.1* expression in Upland cotton leaves could attenuate wax accumulation and lead to enhanced susceptibility to desiccation (Lu et al., 2021).

**TABLE 1:** Summary of plant resilience to drought, salinity, temperature, and ultraviolet (UV) stress contributed by cuticle biosynthesis.

Plant stress resilience traits	Stress resilience-related cuticle biosynthesis gene	Stress resilience- related cuticle components	Plant species	Contribution of cuticle biosynthesis to plant stress resilience and evidence	References
Drought stress resilience	*LACS1*, *LACS2, LACS4*	Cutin and cuticular wax	*Arabidopsis thaliana*, *Malus domestica*, *Glycine max*	*Arabidopsis lacs1 lacs2* double-mutant plants displayed deficiency in cutin synthesis together with high susceptibility to drought stress. Ectopic expression of apple *MdLACS2/4* and soybean *GmLACS2-3* in *Arabidopsis* could enhance the accumulation of wax and cutin respectively, leading to the increased plant drought tolerance.	Weng et al., 2010; Zhang et al., 2020a,b; Ayaz et al., 2021
	*CER1*	Cuticular wax	*A. thaliana*, *Oryza sativa*, *Solanum lycopersicum*, *Triticum aestivum*	Overexpression of *Arabidopsis AtCER1*, wheat *TaCER1-1A*, cucumber *CsCER1*, rice *OsGL1-2 and OsGL1-3* could promote wax alkane biosynthesis and enhance drought tolerance in the transgenic plants. Knockout or knockdown of the tomato *SlCER1-1*, rice *OsGL1-1* and *OsGL1-6* genes all led to drought hypersensitive phenotypes and attenuated cuticle wax alkane accumulation.	Islam et al., 2009; Bourdenx et al., 2011; Qin et al., 2011; Zhou et al., 2013, 2015; Wang et al., 2015; Wu et al., 2022
	*CER2*	Cuticular wax	*M. domestica*	Ectopic expression of apple *MdCER2* in *Arabidopsis* could result in enhanced wax accumulation and increased plant drought tolerance.	Zhong et al., 2020
	*CER9*	Cutin and cuticular wax	*A. thaliana*	*Arabidopsis cer9* mutant plants displayed altered deposition of cuticular cutin and wax together with enhanced plant drought resistance.	Lü et al., 2012; Zhao et al., 2014
	*OSP1*	Cuticular wax	*A. thaliana*	*Arabidopsis osp1* mutants exhibited defects in the formation of stomatal cuticular ledges and enhanced drought tolerance.	Tang et al., 2020
	*DWA1*	Cuticular wax	*O. sativa*	Rice *dwa1* knock-out mutant exhibited attenuated cuticular wax accumulation and high sensitivity to drought stress.	Zhu and Xiong, 2013
	*KCS1*, *KCS2*, *KCS6*	Cuticular wax	*Arachis hypogaea*, *Camellia sinensis*, *M. domestica*,	Overexpression of groundnut *AhKCS1*, apple *MdKCS2*, and orange *CsKCS6* all led to enhanced wax accumulation and drought tolerance in transgenic plants.	Lokesh et al., 2019; Guo et al., 2020; Lian et al., 2021;
	*FAR3.1*	Cuticular wax	*Gossypium hirsutum*	Silencing the *GhFAR3.1* gene in cotton leaves could attenuate wax accumulation and resistance against desiccation.	Lu et al., 2021
	*GPAT2*	Cutin	*Physcomitrella patens*	Disruption of *PpGPAT2* in *P. patens* plant attenuated cutin accumulation and plant drought tolerance.	Lee et al., 2020
	*WSD1*	Cuticular wax	*A. thaliana*	*Arabidopsis wsd1* mutant plants displayed reduced wax ester coverage together with compromised plant drought tolerance, whereas over-expression of *AtWSD1* in *Arabidopsis* and *Camelina* resulted in enhanced drought tolerance in transgenic plants.	Patwari et al., 2019; Abdullah et al., 2021
	*GL6*	Cuticular wax	*Zea mays*	Maize *gl6* mutant exhibited decreased epicuticular wax accumulation and attenuated seedling drought tolerance.	Li et al., 2019
	*ABCG9*, *ABCG31*	Cutin and cuticular wax	*Hordeum vulgare*, *O. sativa*	Loss of function of *HvABCG31* gene in barley led to a deficiency in cutin biosynthesis and plant drought hypersensitivity, whereas rice *osabcg9-2* mutant displayed attenuated wax accumulation and enhanced drought susceptibility.	Chen et al., 2011; Nguyen et al., 2018
	*MYB94, MYB96*	Cutin and cuticular wax	*A. thaliana*, *Z. mays*	Overexpression of Arabidospis *AtMYB94* and *AtMYB96* could enhance the wax accumulation and potentiate drought tolerance in transgenic plants, whereas lack of *ZmFDL1*/*MYB94* in maize led to a reduction in the biosynthesis of cuticular cutin and wax biosynthesis, as well as desiccation hypersensitivity.	Lee et al., 2016; Castorina et al., 2020
	*SHN1, SHN2, SHN3*	Cutin and cuticular wax	*A. thaliana*, *T. aestivum*, *H. vulgare*, *S. lycopersicum*, *M. domestica*	Overexpression of *Arabidopsis AtSHN1/2/3*, barley *HvSHN1*, wheat *TaSHN1*, apple *MdSHINE2* and tomato *SlSHN1* all resulted in the enhanced wax coverage and increased drought resistance in transgenic plants.	Aharoni et al., 2004; Al-Abdallat et al., 2014; Bi et al., 2018; Zhang et al., 2019b; Djemal and Khoudi, 2021;
	*WRI4*	Cuticular wax	*Cyperus esculentus*	Ectopic expression of yellow nutsedge *WRI4-like* gene in *Arabidopsis* resulted in enhanced cuticular wax accumulation and improved plant drought tolerance.	Cheng et al., 2020
	*RAP2.4*	Cuticular wax	*A. thaliana*	Overexpression of *Arabidopsis AtRAP2.4* gene led to enhanced wax accumulation and increased desiccation tolerance in transgenic plants.	Yang S. U. et al., 2020
	*DHS*	Cuticular wax	*O. sativa*	Overexpression of rice *DHS* inhibited wax accumulation and drought tolerance in transgenic plants.	Wang Z. et al., 2018
	*SRL5*	Cuticular wax	*Z. mays*	The maize loss-of-function mutant *srl5* exhibited abnormal wax crystal morphology and distribution, as well as hypersensitivity to drought stress.	Pan et al., 2020
Salinity stress resilience	*WSD1*	Cuticular wax	*A. thaliana*	Overexpression of the wax ester biosynthesis gene *WSD1* in *Arabidopsis* resulted in an increase in leaf and stem wax loading and the enhanced plant tolerance to salinity stress.	Abdullah et al., 2021
	*LACS2, LACS4*	Cuticular wax	*M. domestica*	Ectopic expression of apple *MdLACS2* and *MdLACS4* in *Arabidopsis* resulted in enhanced wax accumulation and salt stress resistance.	Zhang et al., 2020a,b
	*WBC11*	Cutin and cuticular wax	*A. thaliana*	*Arabidopsis* loss-of-function mutant of the *AtWBC11* gene exhibited reduced levels of cutin monomers and wax constituents, as well as decreased tolerance to salinity stress.	Bird et al., 2007
	*SHN1*	Cuticular wax	*H. vulgare*	Ectopic expression of barley *HvSHN1* could enhance wax biosynthesis gene expression and increase salinity tolerance in transgenic tobacco plants.	Djemal and Khoudi, 2021
	*MYB49*	Cutin	*A. thaliana*	Cutin deposition and salt tolerance were enhanced in transgenic *Arabidopsis* plants overexpressing *AtMYB49* but attenuated in transgenic plants overexpressing the chimeric repressor AtMYB49-SRDX49 fusion construct.	Zhang et al., 2020c
	*GPAT2*	Cutin	*P. patens*	Disruption of *PpGPAT2* in *P. patens* plant leads to attenuated cutin deposition together with reduced plant salinity resilience.	Lee et al., 2020
Extreme temperature stress resilience	*SHN1*	Cuticular wax	*H. vulgare*	Ectopic expression of barley *HvSHN1* in tobacco could activate the expression of tobacco wax biosynthesis gene *NtCER1* and potentiate plant heat tolerance.	Djemal and Khoudi, 2021
	*OSCs*	Cuticular wax triterpenoids	*Sorghum bicolor*	Cuticular wax triterpenoids biosynthesis mediated by sorghum SbOSCs contributes to the reinforcement of plant cuticles in a spatial pattern to restrict water loss at high temperatures.	Busta et al., 2021
	*TT2*	Cuticular wax	*O. sativa*	Rice *OsTT2* null mutant displays enhanced retention of wax at high temperatures and increased thermotolerance.	Kan et al., 2022
	*ACC1*	Cuticular wax	*A. thaliana*	A missense mutation in *Arabidopsis AtACC1* attenuated wax deposition on inflorescence stem and resulted in plant supersensitivity to freezing stress.	Amid et al., 2012
	*WXP1, WXP2*	Cuticular wax	*M. truncatula*	Ectopic expression of barrel medic *MtWXP1* in *Arabidopsis* promoted accumulation of wax n-alkanes and primary alcohols, leading to enhanced freezing tolerance, whereas transgenic *Arabidopsis* plants overexpressing *MtWXP2* displayed reduced freezing tolerance accompanied with a decreased level of primary alcohols.	Zhang et al., 2007
	*CER3*	Cuticular wax	*A. thaliana*	Cold-acclimated *Arabidopsis* mutant *cer3-6* exhibited reduced accumulation of wax alkanes and alcohols, and froze at warmer temperatures compared to WT.	Rahman et al., 2021
	*DEWAX*	Cuticular wax	*A. thaliana*	Cold-acclimated *Arabidopsis* mutant *dewax* exhibited enhanced accumulation of wax, and displayed freezing exotherms at colder temperatures compared to WT.	Rahman et al., 2021
UV stress resilience	Not identified	Cuticular phenolics	*S. lycopersicum*	UV-Vis spectrometry analysis showed that cuticle membranes isolated from tomato fruit could screen the UV-B light by 99%, which is mainly attributed to the UV absorption mediated by phenolic acids.	Benítez et al., 2022
	Not identified	Cuticular phenolics	*Capsicum annuum*, *Vitis vinifera*, *Brassica oleracea*, *Beta vulgaris*, *Hedera helix*, *Iris germanica*, *Agave Americana*, *Clivia miniata*	Ultrafast transient spectroscopy analysis revealed that UV-B photoprotection varies from above 99% to more than 50% for the tested cuticle samples isolated from multiple plant species, and the major UV-B attenuation could be attributed to the UV-B absorbance by cuticular phenolic compounds.	González Moreno et al., 2022

Recent studies on transcription factors governing cuticle biosynthesis provide new insight into plant cuticle biosynthesis and drought stress adaptation. For instance, over-expression of *Arabidopsis* transcription factor genes *AtSHN1*, *AtSHN2 and AtSHN3*, as well as their homologs in barley (*HvSHN1*), wheat (*TaSHN1*), apple (*MdSHINE2*) and tomato (*SlSHN1*), could induce expression of wax biosynthesis genes and result in the increased wax accumulation and enhanced plant drought resistance (Aharoni et al., 2004; Al-Abdallat et al., 2014; Bi et al., 2018; Zhang et al., 2019b; Djemal and Khoudi, 2021). Over-expression of *Arabidopsis* transcription factor gene *RAP2.4* could upregulate the expression of wax biosynthesis genes *KCS2* and *CER1*, resulting in increased wax content and enhanced drought tolerance (Yang S. U. et al., 2020). In addition, the rice homeodomain leucine zipper IV (HD-ZIP IV) family of transcription factor ROC4 could positively regulate wax biosynthesis and drought tolerance by directly activating the cuticle biosynthesis gene *OsBDG* (Wang Z. et al., 2018). Interestingly, a RING-type E3 ligase DHS (DROUGHT HYPERSENSITIVE) negatively regulates rice wax biosynthesis and drought tolerance by targeting ROC4 for ubiquitin-mediated proteasomal degradation, suggesting the regulatory role of ubiquitin/26S proteasome (UPS) pathway in the transcriptional reprograming essential for plant cuticle biosynthesis and drought stress adaptation (Wang Z. et al., 2018).

## Cuticle biosynthesis in plant adaptation to salinity stress

As a water loss barrier, the cuticle functions to reduce the transpiration rate and avoid tissue dehydration under salinity stress. Up-regulation of cuticle biosynthesis genes and elevated accumulation of wax and cutin is observed in model and crop plants in response to salinity stress. Increasing evidence revealed that plant tolerance to salinity stress could be enhanced by over-expression of cuticle biosynthesis genes and reduced by knockout or knockdown of cuticle biosynthesis genes (summarized in Table 1). For instance, over-expression of the wax ester biosynthesis gene *WSD1* in *Arabidopsis* could increase leaf and stem wax loading, leading to enhanced plant tolerance to salinity stress (Abdullah et al., 2021). Ectopic expression of *MdLACS2* and *MdLACS4*, apple homologs of *Arabidopsis AtLACS2* and *AtLACS4*, could enhance the wax accumulation and salt stress resistance in transgenic *Arabidopsis* plants (Zhang et al., 2020a,b). Recent studies on transcription factors that regulate cuticle biosynthesis shed novel light on the involvement of plant cuticle biosynthesis in plant salinity stress adaptation. For instance, ectopic expression of *HvSHN1*, barley homolog of *Arabidopsis* transcription factor gene *AtSHN1*, could induce expression of wax biosynthesis gene *NtCER1* and increase salinity tolerance in transgenic tobacco (*Nicotiana tabacum*) plants (Djemal and Khoudi, 2021). Recent transcriptome analysis revealed that the AtMYB49 could directly activate *MYB41*, *ASFT*, *FACT*, and *CYP86B1*, representative genes in the category of ‘cutin, suberin, and wax biosyntheses’ (Zhang et al., 2020c). The biochemical analysis further showed that cutin deposition and salt tolerance were higher in transgenic *Arabidopsis* plants overexpressing AtMYB49 but lower in transgenic plants overexpressing the chimeric repressor AtMYB49-SRDX49 fusion construct, suggesting that transcriptional activation of cutin biosynthesis by AtMYB49 contributes to the plant salt tolerance in *Arabidopsis* (Zhang et al., 2020c).

## Cuticle biosynthesis in plant adaptation to extreme-temperature stress

Since the heat capacity of the cuticle was above the one of air, the cuticle could function as a heat sink to impair the heat transference between plant aerial organs and the surrounding environment, thereby governing the temperature increment in plant tissues at high temperatures. Differential scanning calorimetry (DSC) measurements showed that high-temperature stress could induce wax melting and glass transition, as well as increased permeability, in tomato fruit cuticle membrane (Benítez et al., 2022). As summarized in Table 1, molecular genetic studies of cuticle biosynthesis genes shed more light on plant adaptation to heat stress. A recent cuticular wax chemical analysis showed that triterpenoids enriched in adult leaf waxes of *S. bicolor*, a heat and drought-tolerant crop, but not its close relative *Z. mays* (maize) (Busta et al., 2021). Further comparative genomics and heterologous expression analyses revealed that sorghum cuticular triterpenoids are synthesized by a neofunctionalized steroid biosynthesis gene that is truncated and not expressed in maize, which leads to the reinforcement of sorghum cuticle in a spatial pattern to restrict water loss at high temperatures (Busta et al., 2021). In addition, a natural quantitative trait locus (QTL), *TT2* (*THERMOTOLERANCE 2*), that could confer thermotolerance in rice without a yield penalty was recently found to encode a Gγ subunit that regulates the heat-triggered elevation of Ca^2+^, which leads to the activation of OsSCT1 (Sensing Ca2 + Transcription factor 1) protein (Kan et al., 2022). Interestingly, OsSCT1 was revealed to function as a negative regulator of cuticle biosynthesis genes such as the transcription factor gene *OsWR2*. Therefore, a natural loss-of-function allele *TT2* fails to initiate the heat-triggered elevation of Ca^2+^, which results in the inactivation of SCT1 and de-repression of *OsWR2* at high temperature, thereby inducing the wax biosynthesis and conferring the rice thermo-tolerance (Kan et al., 2022).

Activation of cuticle biosynthesis, such as up-regulation of cuticle biosynthesis genes, by cold stress has been observed in model and crop plants such as *A. thaliana*, *M. truncatula*, *T. aestivum*, *B. distachyon*, *Thellungiella salsuginea*, and *Cucurbita pepo* (Lee and Suh, 2015; Wang Y. et al., 2018; He et al., 2019). Interestingly, overaccumulation of cuticular wax alkanes was induced by cold stress in fruits of cold-tolerant zucchini variety “Natura,” but not the cold-sensitive variety “Sinatra,” suggesting the potential contribution of the wax alkane biosynthesis to the postharvest quality improvement of zucchini fruit during low-temperature storage (Carvajal et al., 2021). Current evidence revealed that plant tolerance to cold stress is affected by altered expression of cuticle biosynthesis genes (summarized in Table 1). For instance, Cold-acclimated *Arabidopsis* mutant *cer3-6* exhibited reduced accumulation of wax alkanes and alcohols, and froze at warmer temperatures compared to WT. In contrast, cold-acclimated *dewax* mutant exhibited enhanced accumulation of wax alkanes and alcohols, and displayed freezing exotherms at colder temperatures compared to WT, indicating that cuticular wax contributes to plant tolerance to cold stress.

## Cuticle biosynthesis in plant adaptation to ultraviolet radiation stress

Due to stratospheric ozone depletion during past decades, solar UV radiation that reached the earth surface has increased, especially in the Arctic and Northern biosphere. Based on the wavelength region, UV radiation could be divided into UV-A (315–400 nm), UV-B (280–315 nm), and UV-C (200–280 nm) (Robson et al., 2019). Through enhancing the generation of reactive oxygen species (ROS) to damage macromolecules such as DNA, proteins and lipids, energetic UV-B is harmful to land plants (Robson et al., 2019). Upon exposure to the UV-B radiation, increases in cuticle thickness and wax deposition have been reported in multiple plant species such as *Cucumis sativus*, *P. vulgaris*, *H. vulgare*, *Coffea arabica*, and *C. canephora*. As major UV-screening compounds in land plants, phenolic acids, and flavonoids have been identified in the cuticular wax fraction, cutin matrix and even cell walls. Current evidence showed that flavonoids could function as UV-A attenuators, whereas phenolic acids mainly contribute to the UV-B and UV-C photoprotection. Indeed, UV-Vis spectra analysis revealed that cuticle membranes isolated from tomato fruit could screen the UV-B light by 99%, which is mainly attributed to the UV absorption mediated by phenolic acids but not cuticular waxes (Benítez et al., 2022). Ultrafast transient spectroscopy analysis showed that the UV-B photoprotection varies from above 99% to more than 50% for the tested cuticle samples isolated from multiple plant species, and the major UV-B attenuation could be attributed to the UV-B absorbance by cuticular phenolic compounds (González Moreno et al., 2022). Quantum chemical computational analyses further revealed that UV-B energy absorbed by cuticular phenolic compounds can be either released as blue fluorescence via the radiative mechanism or dissipated via the conformational isomerization of phenolic compounds (González Moreno et al., 2022).

## Harnessing cuticle biosynthesis for crop adaptation to environmental stress

Environmental stresses such as drought, salinity, extreme temperatures, and UV radiation adversely affect plant growth and crop production, which has become increasingly serious under stratospheric ozone depletion and global climate change. As an adaptive innovation in land plants, hydrophobic cuticle shields plant tissues from environmental stresses associated with terrestrialization and represents a valuable resource for crop improvement. Cuticle-associated traits have been employed in traditional crop breeding (Petit et al., 2017). For instance, glaucousness determined by epicuticular wax deposition has been selected as a target trait for crop drought tolerance in traditional breeding. Indeed, a recent study revealed that increased leaf wax *n*-alkane concentration has been selected for enhanced productivity in five wheat cultivars developed over the past half-century (Liu et al., 2019). In addition, advanced breeding strategies and approaches established in recent years have paved the new path for harnessing cuticle biosynthesis to improve crop adaptation to stressful environments (Bailey-Serres et al., 2019; Varshney et al., 2020; Gao, 2021).

Cuticle-associated genetic variants have been isolated from natural populations in multiple crop species such as *Z. mays*, *O. sativa*, *S. lycopersicum*, *C. sativus*, and *B. napus* through visual screen for altered reflectance (glaucousness, glossiness, or bloom) of plant aerial organs. As summarized in prior reviews, a set of phenotyping techniques such as toluidine blue soaking, gas chromatography coupled with mass spectrometry (GC-MS), gas chromatography equipped with flame ionization detector (GC-FID), Fourier transform infrared (FTIR) imaging, and spectroscopy, matrix-assisted laser desorption/ionization (MALDI) imaging and leaf radiometric measurements have been developed to analyze cuticle properties, chemical composition and physical structure, which greatly contribute to the characterization and high-throughput phenotyping of cuticle-related genetic variants in crop plants (Petit et al., 2017; Camarillo-Castillo et al., 2021). In addition to natural genetic diversity, cuticle-associated genetic variants have been obtained from the induced mutant population of crop plants *Z. mays*, *O. sativa*, *S. lycopersicum*, and *H. vulgare*. Chemical, irradiation and insertion mutagenesis methods are available for generating the induced mutant populations. EMS (ethyl methanesulfonate) chemical mutagenesis and irradiation mutagenesis with x/y-rays or fast neutrons are widely employed to generate induced mutation in commercial crop breeding. At the same time, T-DNA insertion mutant populations have been constructed in model and crop plants, including *A. thaliana*, *Brachypodium distachyon*, *Z. mays*, *S. lycopersicum*, and *O. sativa*, from which cuticle-associated genetic mutants were identified (Choi et al., 2022).

Map-based cloning of cuticle-associated genetic mutation leads to the identification of cuticle genes in model and crop plants, which has been extensively discussed in previous reviews. Although many cuticle genes identified in the model plant *Arabidopsis* control cuticle-associated traits in a Mendelian manner, cuticle-associated traits in natural crop populations are usually controlled by quantitative trait loci (QTLs). Conventional QTL mapping and genome-wide association studies (GWAS) were employed for identifying these cuticle-related QTLs in crop species. For instance, the natural QTL *TT2* (*THERMOTOLERANCE 2*) conferring rice thermotolerance was identified by QTL mapping as a G-protein γ subunit gene controlling cuticular wax biosynthesis in rice plants (Kan et al., 2022). Similarly, genome-wide association study of natural variation for maize leaf cuticular conductance identifies cuticle genes associated with enhanced crop productivity under drought stress. Through employing the combined chemical analyses, heterologous expression, and comparative genomics, Busta et al. (2021) demonstrated that a neofunctionalized steroid biosynthesis gene that is truncated and not expressed in maize is essential to the biosynthesis of cuticular triterpenoids and thermotolerance in sorghum plants, suggesting that combined metabolomic, transcriptomic, and genomic analyses contribute to the identification of cuticle genes. These cuticle-related QTLs can be used as makers in crop genomic breeding (GB) to facilitate the marker-assisted selection (MAS) and marker-assisted backcrossing (MABC) (Varshney et al., 2020). Through combining chemical or irradiation mutagenesis with genome-wide screening, targeting induced local lesions in genomes (TILLING) could effectively induce mutation in cuticle-related QTLs and genes, and represents a promising non-transgenic method for improving cuticle-associated traits in crop species. For instance, a drought-insensitive rice mutant (*ditl1*) harboring a mutation in the *LOC_Os05g48260* gene, a putative rice ortholog of *WSD1*, was selected by drought stress screening in the rice TILLING population (Choi et al., 2022).

As summarized in Table 1, genetic engineering of cuticle genes was widely employed for improving crop tolerance to drought, salinity and extreme temperature stress. For instance, overexpression of groundnut wax biosynthesis gene *AhKCS1* led to enhanced leaf epicuticular wax accumulation and increased drought tolerance in transgenic groundnut plants (Lokesh et al., 2019). Overexpression of wheat transcription factor gene *TaSHN1* and tomato *SlSHN1* resulted in the enhanced wax coverage and increased drought resistance in transgenic wheat and tomato plants, respectively (Al-Abdallat et al., 2014; Bi et al., 2018). In addition, ectopic expression of cuticle genes identified from model and crop plants was conducted to improve crop stress tolerance. For instance, ectopic expression of *Arabidopsis AtMYB96* and *AtWSD1* in Camelina plants could potentiate plant drought tolerance (Lee et al., 2014; Abdullah et al., 2021). Similarly, ectopic expression of wheat cuticle gene *TaCER1-1A* in rice plants could promote rice wax alkane biosynthesis and enhance rice leaf tolerance to desiccation stress (Li et al., 2019). For the biosafety of transgenic crops, many marker-free transgenic approaches have been established. Cao et al. (2020) reported the development of marker-free and transgene insertion site-defined (MFTID) transgenic wheat lines with improved grain storability and fatty acid content through suppressing *lipoxygenase* (*LOX*) gene expression in wheat grains, which provides a new avenue for the genetic engineering of cuticle genes in crop species.

Through using sequence-specific nucleases (SSNs), plant genome editing (GE) could introduce precise DNA mutations such as insertion, deletion and base substitution into target genome regions (Manghwar et al., 2019; Gao, 2021). As the most recently developed GE technique, CRISPR (clustered regularly interspaced short palindromic repeats)-Cas9 (CRISPR associated nuclease 9) system relies on the programmable guide RNA (gRNA) to guide the Cas9 nuclease to the DNA targets, where DNA double-strand breaks (DSBs) are generated and precise GE is achieved via endogenous DNA repair pathways (Manghwar et al., 2019; Gao, 2021). CRISPR-Cas9 system has been successfully applied in generating genome-edited crop plants with altered cuticle traits. For instance, precise editing of the *OsPYL9* gene, one of the ABA receptor genes in rice, by CRISPR-Cas9 system resulted in the overaccumulation of cuticular wax and enhanced drought tolerance in genome-edited rice plants (Usman et al., 2020). Similarly, multiplex knockout of transcription factor gene *MYB186* and its paralogs *MYB138* and *MYB38* in hybrid poplar by CRISPR-Cas9 system with a single gRNA led to a glabrous phenotype accompanied with the absence of wax triterpenes in trichomeless leaves (Bewg et al., 2022). These studies paved a new path for generating genome-edited crop plants with improved cuticle traits and enhanced stress tolerance in future research.

## Concluding remarks and perspectives

**FIGURE 1:**
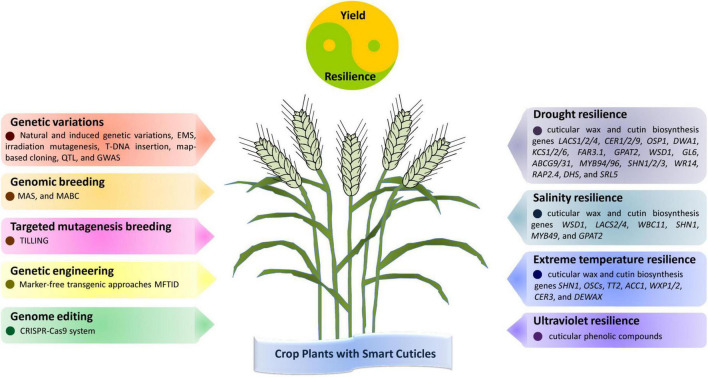
Strategies and targets in harnessing cuticle biosynthesis for crop resilience to drought, salinity, temperature, and ultraviolet (UV) stress. Cuticle-related genetic variations could be either identified from natural populations or artificially induced, which would facilitate crop breeding for cuticle-associated traits through genomic breeding. In addition, crop plants with improved resilience to drought, salinity, temperature, and UV stress could be generated by targeted mutagenesis, genetic engineering, and genome editing of cuticle biosynthesis genes. These crop plants with smart cuticles would display improved performance in yield and resilience under drought, salinity, temperature, and UV stress.

In this review, we highlighted recent advances in cuticle biosynthesis and its roles in plant adaptation to drought, salinity, extreme temperatures, and UV radiation stress, and discussed the current strategies and future directions in harnessing cuticle biosynthesis for crop improvement. As shown in Figure 1, cuticle-related genetic diversity could be either identified from natural populations or artificially induced, which would facilitate crop breeding for cuticle-associated traits through breeding approaches MAS and MABC. In addition, crop plants with improved cuticle-related traits and stress tolerance could be generated by non-transgenic TILLING, genetic engineering and genome editing of cuticle biosynthesis genes. Although the past decade has seen great progress in the molecular biology of plant cuticle biosynthesis, we still have a long way to go toward fully understanding the mechanism of cuticle biosynthesis in plant adaptation to drought, salinity, extreme temperatures, and UV radiation stress, as well as its application in crop improvement. For instance, most of our knowledge about plant cuticle biosynthesis comes from the study of model plants *Arabidopsis*, mechanisms of cuticle biosynthesis in important crops remain to be explored. Furthermore, the expression of some cuticle biosynthesis genes could be induced by environmental stress, but the underlying mechanisms of cuticle biosynthesis responding to environmental stress remains to be disclosed. Moreover, cuticle generally shields plant tissues from environmental stress, but the exact roles and mechanisms of cuticle structure and components in plant adaptation to specific environmental stress are poorly understood. In addition, overexpression of cuticle biosynthesis gene usually enhances crop stress resilience with a yield penalty due to the altered metabolic flux allocation, breeding crop variety with a ‘smart cuticle’ that confer crop plants improved stress tolerance at a low cost of energy and metabolic flux might be essential to balancing crop yield and resilience. With the advance in the knowledge of plant cuticle biosynthesis in plant adaptation to drought, salinity, extreme temperatures and UV radiation stress, generating this ‘smart cuticle’ with improved structure and optimized composition would certainly provide new avenues for crop improvement under adverse environments.

## Author contributions

CC, LL, and XW wrote the manuscript. All authors have read and agreed to the published version of the manuscript.
